# Oral administration of ethanol extract from *Gynura procumbens* stems corrects kidney injury and renal anemia in chronic kidney disease

**DOI:** 10.3389/fphar.2024.1476735

**Published:** 2025-01-08

**Authors:** Ting-Ting Li, Li-Ying Wen, Sha-Sha Meng, Yu-Sang Li, He-Bin Tang

**Affiliations:** Lab of Hepatopharmacology and Ethnopharmacology, School of Pharmaceutical Sciences, South-Central Minzu University, Wuhan, China

**Keywords:** chronic kidney disease, renal anemia, ethanol extract from *Gynura procumbens* stems, chlorogenic acid, trans-p-coumaric acid

## Abstract

**Background:**

*Gynura procumbens* (Lour.) Merr. is a plant used in traditional Chinese medicine that reduces hepatotoxicity, relieves kidney discomfort, and has anti-inflammatory and antioxidant properties.

**Methods:**

We aimed to explore the mechanisms underlying the therapeutic effects of an ethanol extract from *G. procumbens* stems (EEGS) and selected metabolites on kidney injury and renal anemia associated with chronic kidney disease (CKD). An adenine-induced rat CKD model was used to elucidate the effective mechanism of EEGS and selected metabolites to correct renal anemia.

**Results:**

The results showed that treatment with EEGS reversed abnormal changes in the blood indicators, including hemoglobin, red blood cells, serum erythropoietin (EPO), and creatinine levels. Moreover, EEGS inhibited xanthine oxidase (XOD) activity *in vitro*, significantly inhibited liver XOD activity, and reduced intrahepatic inflammatory infiltration. Analysis of the pathological changes revealed that EEGS treatments resulted in reduced renal tubular apoptosis, decreased a number of crystals, a narrowed tubular lumen, and attenuated tubular fibrosis. Immunohistochemical staining revealed that EEGS significantly ameliorated the adenine-induced abnormal changes in the expression of related proteins, including β-catenin, COX-2, HIF-2α, and EPO, in the rat kidney tissues. Among the selected EEGS metabolites, the combined effect of chlorogenic acid and trans-*p*-coumaric acid was superior to that of either compound alone.

**Conclusion:**

These results suggest that EEGS and selected metabolites can effectively correct renal anemia in CKD rats by inhibiting XOD activity, reducing inflammation, and alleviating renal injury.

## 1 Introduction

The kidneys play an important role in regulating erythropoiesis. When structural damage to the kidneys persists, or when kidney dysfunction evolves slowly, it can occur renal anemia. Renal anemia, a common complication of chronic kidney disease (CKD), has been a major challenge in the management of clinical kidney disease (Zadrazil and Horak, 2015). Despite significant advancements in the understanding and treatment of anemia in CKD patients over the past few decades, the high prevalence of anemia among patients, and its adverse effects on quality of life, remain of considerable concern (Ku et al., 2023). Anemia makes patients susceptible to fatigue and weakness and exacerbates the deterioration of renal function, in addition to causing neurocognitive deficits and an increased risk of cardiovascular events (Mei et al., 2021).

In recent years, increasing research has suggested that renal anemia is not solely attributable to impaired hematopoietic function but is closely associated with the pathophysiological changes inherent to kidney disease itself (Portolés et al., 2021). Kidney injury is caused by damaged or abnormal kidney tissues or function and usually caused by a chronic persistent disease or acute pathology. Chronic renal tissue damage results in hematopoietic dysfunction, which often manifests itself in the development of anemia over time. Adenine, an inducer of renal anemia in CKD, primarily involves damage to the kidneys (Wu et al., 2019). Xanthine oxidase (XOD), a critical enzyme that catalyzes the conversion of adenine to uric acid, is highly active, resulting in the deterioration of renal function and accelerated progression of CKD (Balakumar et al., 2020). Studies indicate that XOD inhibitors are receiving considerable research attention as a potential therapeutic approach, not only for their efficacy in lowering hyperuricemia but also for their potential to improve renal function by directly intervening in the physiological processes of the kidneys.

Additionally, anemia associated with CKD is a specific form of inflammatory anemia, characterized by the release of cytokines in response to inflammation, which causes progressive loss of renal function and impaired erythropoietin (EPO) synthesis (Olivari et al., 2023). EPO is a driver that primarily maintains erythrocyte survival and plays a key role in renal anemia. Normally, EPO secreted by the kidneys can stimulate the bone marrow to produce sufficient erythropoiesis. However, when the kidneys are damaged, a decrease in EPO production leads to insufficient erythropoiesis in the blood, which triggers renal anemia. This series of pathological and physiological changes ultimately allows kidney injury to develop into chronic kidney disease. Evidence shows that after the inflammatory stimulus subsides, EPO inhibits apoptosis and renal fibrosis and returns to its original characteristics (Souma et al., 2016). In the case of hypoxia, hypoxia-inducible factor 2α (HIF-2α) primarily regulates EPO, which in turn optimizes and maintains tissue oxygen homeostasis (Brines and Cerami, 2008).

Currently, therapeutic strategies for renal anemia focus on correcting anemia itself through the use of erythropoiesis-stimulating agents, recombinant human EPO, and inhibitors of prolyl-hydroxylase-HIF, which are used primarily to increase EPO levels and stimulate erythropoiesis (Heras-Benito, 2023). However, the therapeutic efficacy of these treatments is limited, and their long-term use may increase the risk of thrombosis (Yu et al., 2021). Therefore, safer and more effective alternative treatments are urgently needed.

*Gynura procumbens* (Lour.) Merr., commonly known as long-life spinach, is widely grown in Southeast Asia. It is traditionally used to relieve kidney complaints and treat kidney diseases (Tan et al., 2016). In southern China, *Gynura procumbens* is consumed as a vegetable or used medicinally to treat rheumatic arthralgia and gout. In addition, *G. procumbens* can be used to prevent bleeding and replenish blood, making it a potentially effective drug for improving renal anemia (< Chinese Materia Medica > edited by the State Administration of Traditional Medicine of China; Goverment, 2005). Abnormal stimulation of the Wnt/β-catenin signaling pathway worsens renal injury and exacerbates anemia (Zhao et al., 2019). Our earlier investigation revealed that EEGS can attenuate the inflammatory response by inhibiting the expression of β-catenin and COX-2 (Zhang et al., 2022). Moreover, we identified chlorogenic acid (CA) and trans-*p*-coumaric acid (t-*p*-CA) as critical active constituents of EEGS that have anti-inflammatory and antioxidant properties (Wang et al., 2022). Koriem et al. reported that CA can effectively treat anemia induced by octylphenol and improve hepatic and renal functions (Koriem, Arbid, and Gomaa, 2018). T-*p*-CA is known to promote the absorption of CA and maximize its therapeutic efficacy (Wang et al., 2022). Considering these previous findings, herein, we investigated the therapeutic mechanism of EEGS in treating renal anemia.

## 2 Materials and methods

### 2.1 Drugs and reagents

The whole *G. procumbens* plant (Hainan, China) used in this study was identified by Prof. Bing-kun Zhang from the Wuhan Institute of Botany (specimen voucher no. 20180927). EEGS and selected metabolites [chlorogenic acid (13.6%) and trans-*p*-coumaric acid (0.6%), identified in the EEGS by high-performance liquid chromatography] were derived from the samples reported in our previous study, which are now deposited in the sampling room of the Laboratory of Hepatopharmacology and Ethnopharmacology at the School of Pharmacy Sciences, South-Central Minzu University (Wang et al., 2022). EEGS refers to the extract obtained by filtering, concentrating, and drying a mixture of dried stems (0.5 kg) of *G. procumbens* that had been crushed in 5 L of 80% ethanol solution (Li et al., 2015).

The following drugs were used: adenine (Gracia Chemical Technology Co. Ltd., Chengdu, China); allopurinol tablets (Xinyi Wanxiang Pharmaceutical Co. Ltd., Shanghai, China); CA and t-*p*-CA (Yuanye Biological Co., Ltd., Shanghai, China); allopurinol and xanthine oxidase (XOD; Pumeike Biotechnology Co., Ltd., Wuhan, China). The analysis kits used included enzyme-linked immunosorbent assay (ELISA) kits (Jianglai Industrial Limited by Share Ltd., Shanghai, China), creatinine (Cr) kits (Yuanye Bio-Technology Co., Ltd.), XOD test kits (Jiancheng Bioengineering Institute, Nanjing, China), and a TUNEL Cell Apoptosis Detection Kit (Servicebio Technology Co., Ltd., Wuhan, China). The study utilized antibodies against β-catenin, COX-2, HIF-2α, and EPO (Abcam Inc., Cambridge, United Kingdom).

### 2.2 Animal care

The Hubei Experimental Animal Center in China provided us with 40 female Wistar rats (aged 6 weeks, weighing 180–220 g). The rats were acclimatized and fed under specific pathogen-free circumstances for 7 days prior to the start of the experiments. During the experiment, the animals were fed a conventional chow diet and kept in a room with the following conditions: 20°C–25°C, 12 h dark-light cycle, and 60%–70% relative humidity. All animal experiments were approved by the Animal Ethics Committee of South-Central Minzu University (permit number: 2021-SCUEC-044).

**FIGURE 1:**
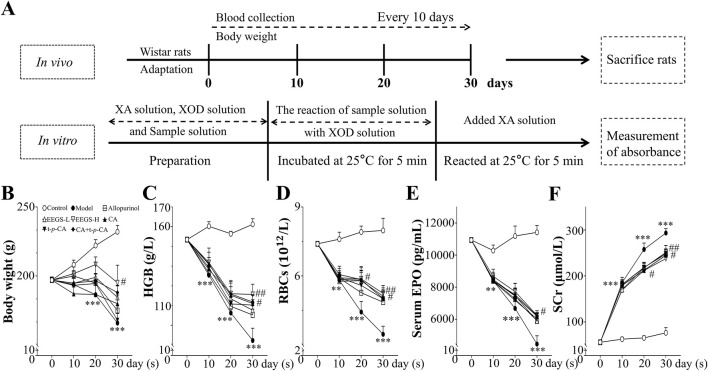
Overview of rats with different treatments. **(A)** Schematic representation of experimental procedures *in vivo* and *in vitro*; **(B)** Effects of administration of EEGS and selected metabolites on body weight of rats; **(C)** Effects of EEGS and selected metabolites on HGB levels before and after the intervention; **(D)** Effects of EEGS and selected metabolites on RBCs before and after the intervention; **(E)** Effects of EEGS and selected metabolites on serum EPO levels before and after the intervention; **(F)** Effects of EEGS and selected metabolites on SCr levels before and after the intervention. Data are expressed as mean ± SEM (n = 5). **p < 0.01, ***p < 0.001 versus control group; ^#^ *p* < 0.05, ^##^ *p* < 0.01 versus adenine group.

For the animal experiments, female Wistar rats were randomly split into eight groups (n = 5 per group): control (equal volume of saline), adenine (250 mg/kg/day), allopurinol tablets (5.138 mg/kg/day), EEGS-L (5 mg/kg/day), EEGS-H (125 mg/kg/day), CA (10 mg/kg/day), t-*p*-CA (10 mg/kg/day) and CA + t-*p*-CA (10 mg/kg/day +10 mg/kg/day, CA and t-*p*-CA mixed at a ratio of 1:1). The rats in all the groups were fed standard chow diet. Adenine (250 mg/kg/day) was administered via oral gavage for 30 consecutive days to the adenine and treatment groups (Wang et al., 2022). The weight of each rat was measured on days 0, 10, 20, and 30 (Figure 1A).

### 2.3 Sample collection and hematological analyses

Blood samples were collected via the tail vein of the rats on days 0, 10, 20, and 30, and hemoglobin (HGB) levels were measured using a hemoglobinometer. Red blood cell (RBC) counts were measured via microscopic counting. Serum EPO levels were measured using an enzyme-linked immunosorbent assay (ELISA) kit, and serum creatinine (SCr) levels were measured using a creatinine (Cr) kit (deproteinized endpoint microplate method). All the rats were sacrificed at the end of 30 days with 100% carbon dioxide, the liver and kidneys were removed and washed with saline, and the volume of the each kidney was measured. After weighing, a portion of the liver tissue was used to determine XOD activity using an XOD assay kit (colorimetric method), while the remainder was used for paraffin sectioning. The liver index was calculated as follows: (liver weight/body weight × 100%). The kidney index was calculated in the same way.

### 2.4 Evaluating the XOD inhibitory effect *in vitro*

The XOD inhibitory activity assay was performed as shown in Figure 1A. In a 96-well ELISA plate, we established sample wells (EEGS, allopurinol, CA, t-*p*-CA, and CA + t-*p*-CA), positive control wells [phosphate-buffered saline (PBS) and XOD solution], negative control wells (PBS), and control wells (PBS and sample solution). The total volume of the reaction mixture was 200 μL. A multimode microplate reader was used (Eastwin Life Science, Inc., Guangzhou, China). The mixture was pre-incubated for 5 min at 25 °C, and the increase in absorption increments at 290 nm was monitored, indicating the formation of uric acid. The xanthine solution was subsequently added to initiate the reaction at 25 °C for 15 min. At 290 nm, the absorbance of the sample wells was calibrated to zero with respect to the absorbance of the control wells to obtain A0 (Khan, 2004), and the absorbance of the positive control wells was zeroed with respect to the absorbance of the negative control wells to obtain A1. The percent inhibition ratio (%) was calculated using the following equation:

$$\text{Inhibition ratio }\left(\%\right)=\left[\left(A1–A0\right)/A1\right]\times 100\%$$

The concentration required for 50% inhibition was calculated as the IC_50_ value (Nguyen et al., 2004). Three replicates were performed for each sample.

### 2.5 Histopathology

Rat liver and kidney tissues were fixed in formalin for 24 h, dehydrated, embedded in wax blocks, and cut into 3-μm sections. Hematoxylin and eosin (H&E) staining was used to assess the pathological morphology of the tissues. The inflammatory scoring criteria for the liver were defined using a ×40 objective lens as follows: 0, 1, 2, and 3 lesions = 0, 1, 2, and 3 points, respectively (Krishna, 2021). Additionally, Masson and TUNEL staining was performed on kidney tissue sections to observe pathological renal damage. The crystalline area, number of crystals, tubular dilatation area, and collagen fiber area were analyzed using ImageJ software (National Institutes of Health, United States). The results are expressed as the percentage of positively stained areas (Rangan and Tesch, 2007).

### 2.6 Immunohistochemistry

The paraffin-embedded sections were soaked in xylene, dewaxed, dehydrated using an alcohol gradient, and incubated with 3% hydrogen peroxide. The sections were subsequently heated in a microwave oven for 10 min in a 0.01 M citrate buffer (pH 6.0). The sections were sealed in an airtight container and incubated with 5% bovine serum albumin (BSA) for 1 h at 37°C; then, the sections were washed with PBS and incubated overnight with anti-EPO, anti-β-catenin, anti-COX-2, or anti-HIF-2α primary antibodies (dilution ratio of 1:200). After washing with PBS, the corresponding secondary antibodies were added to the sections and incubated for 1 h at 37°C, before being stained with diaminobenzidine chromophores. After positive staining, the nuclei were stained with hematoxylin and allowed to return to a blue color in warm water for approximately 5 min. The color was divided using 75% hydrochloric acid–ethanol, and the color returned to blue in warm water. The samples were subsequently dehydrated by immersion in gradient ethanol and sealed by soaking in xylene for clarity. In addition, the TUNEL Cell Apoptosis Detection Kit was used to detect the number of apoptotic cells in the kidneys, following the manufacturer’s instructions. The corresponding multispectral images and quantitative results were acquired and analyzed using Nuance Multispectral Imaging Systems (Cambridge Research & Instruments, MA, United States; Li et al., 2012).

### 2.7 Statistical analysis

All experimental results are shown as the mean ± SEM. The data were analyzed and statistically plotted using Prism 9 software (GraphPad Software, MA, United States). One-way and two-way analysis of variance (ANOVA) tests were conducted with Tukey’s honestly significant difference, and *p* < 0.05 was considered significant.

## 3 Results

### 3.1 EEGS and selected metabolites upregulated adenine-induced reductions in the body weight of the rats and reversed abnormal changes in the blood indicators, including HGB, RBC, serum EPO, and creatinine levels

Oral adenine administration caused a considerable decrease in the body weight of the treated rats after 30 days (Figure 1B). The rats in the adenine group had considerably lower body weights (165.9 ± 6.9 g; *p* < 0.001) than did the control rats (232.0 ± 4.6 g). The EEGS-H treatment showed the best efficacy (195.1 ± 12.7 g; *p* < 0.05). Compared with treatment with either compound alone, CA + t-*p*-CA treatment resulted in greater body weight gain.

As shown in Figures 1C–E, the HGB, RBC, and serum EPO levels of the rats in the adenine group (HGB: 102.3 ± 6.2 g/L, RBCs: 3.5 ± 0.4 ×10^12^/L, serum EPO: 4453.7 ± 519.2 pg/mL; *p* < 0.001) were significantly lower than those in the control group (HGB: 161.2 ± 2.8 g/L, RBCs: 8.0 ± 0.5 ×10^12^/L, serum EPO: 11,420.0 ± 423.6 pg/mL). In the treatment groups, different concentrations of EEGS and selected metabolites increased the HGB levels, RBC counts and serum EPO levels. In particular, the EEGS-H treatment had the best intervention effect compared with the adenine group, with significant increases in the HGB levels (125.6 ± 5.2 g/L; *p* < 0.01), RBC counts (5.2 ± 0.2 × 10^12^/L; *p* < 0.01), and serum EPO levels (6242.5 ± 174.3 pg/mL; *p* < 0.05). The intervention effect was not significant in the allopurinol group.

Adenine-induced rats presented decreased renal function. SCr levels in the adenine-treated rats were considerably elevated beginning on Day 10 and pecked on Day 30 (Figure 1F). Compared with the control group (76.4 ± 11.3 μmol/L), the adenine group presented significantly greater SCr level (293.5 ± 9.6 μmol/L; *p* < 0.001). Compared with the adenine treatment, the EEGS treatment significantly decreased SCr level (237.7 ± 15.5 μmol/L; *p* < 0.01). Furthermore, treatment with CA, t-*p*-CA, and their mixture also significantly lowered SCr levels (245.1 ± 13.5 μmol/L, 247.9 ± 10.9 μmol/L, and 244.8 ± 21.4 μmol/L, respectively; *p* < 0.05), indicating that EEGS and selected metabolites could ameliorate renal damage.

### 3.2 Evaluation of the XOD inhibitory effect of EEGS and selected metabolites *in vitro*

We performed *in vitro* experiments to observe the inhibitory potential of EEGS and selected metabolites on XOD activity. The inhibition rate of each XOD sample increased as the concentration increased to the same final concentration (Table 1). Each sample was active and inhibited by more than 50% at 25.00 μg/mL. At a concentration of 12.50 μg/mL, EEGS and allopurinol inhibited XOD by 50%. EEGS significantly suppressed XOD activity (88.53% inhibition at 100.00 μg/mL), with an IC_50_ value of 11.26 μg/mL. Allopurinol showed 93.56% inhibition at 100.00 μg/mL, with an IC_50_ value of 6.32 μg/mL.

**TABLE 1:** Inhibition rate and IC_50_ of XOD by EEGS and selected metabolites *in vitro*.

Sample	Percentage xanthine oxidase inhibition								IC_50_ (μg/mL)
	0.78 μg/mL	1.56 μg/mL	3.13 μg/mL	6.25 μg/mL	12.50 μg/mL	25.00 μg/mL	50.00 μg/mL	100.00 μg/mL	
Allopurinol	12.68 ± 1.84	25.70 ± 4.63	39.84 ± 2.91	43.86 ± 1.65	67.54 ± 2.21	73.17 ± 0.81	83.97 ± 2.70	93.56 ± 1.25	6.32
EEGS	12.57 ± 0.77	18.76 ± 1.38	26.72 ± 4.03	34.92 ± 0.95	51.61 ± 3.72	63.30 ± 3.65	79.61 ± 1.63	88.53 ± 1.28	11.26
CA	14.87 ± 3.01	21.41 ± 2.84	24.92 ± 2.20	33.12 ± 2.22	40.18 ± 0.79	57.87 ± 2.16	64.31 ± 1.07	82.90 ± 1.27	16.57
t-*p*-CA	11.75 ± 1.56	15.50 ± 2.36	18.87 ± 1.37	26.71 ± 2.45	29.81 ± 4.17	50.56 ± 4.15	65.80 ± 4.97	82.33 ± 1.06	22.79
CA + t-*p*-CA	11.03 ± 1.70	17.02 ± 3.64	23.77 ± 2.23	31.36 ± 0.74	39.70 ± 0.83	59.96 ± 1.18	76.07 ± 1.71	84.53 ± 0.83	15.10

Values are mean ± SEM, of three parallel measurements.

### 3.3 The EEGS and its selected metabolites ameliorated adenine-induced liver injury in rats

**FIGURE 2:**
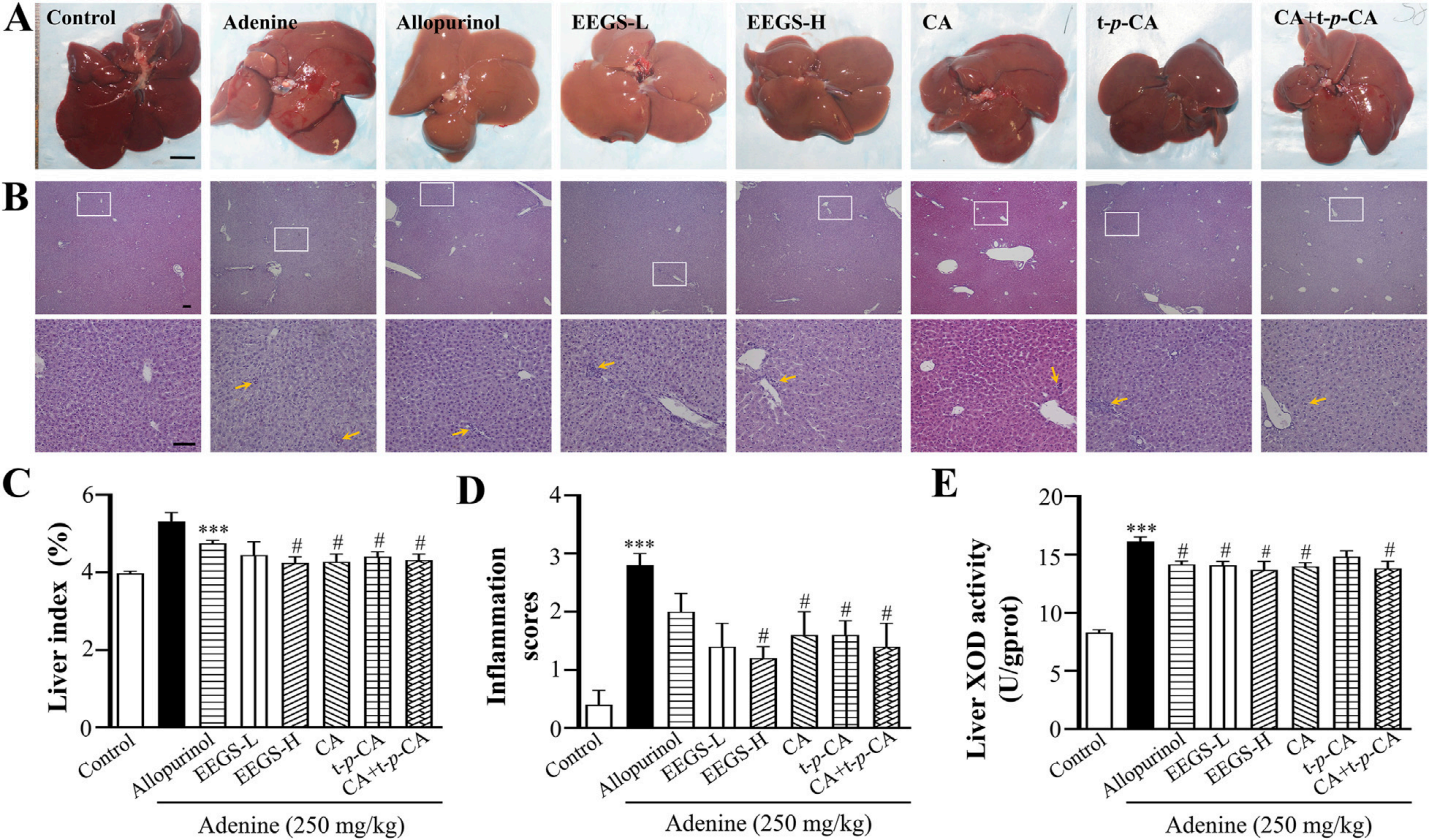
Effects of EEGS and selected metabolites intervention on liver tissue of adenine-induced rats. **(A)** General view of the liver of each group (scale bars: 1 cm); **(B)** H&E staining images of each group (×4 and ×20 objective lens). The orange arrows indicate inflammatory cell aggregation. The second row (×20 objective lens, scale bars: 100 μm) is a partially enlarged view of the first-row picture (×4 objective lens, scale bars: 100 μm); **(C)** Liver index of rats in each group; **(D)** Inflammation scores of liver samples in H&E staining images; **(E)** The activity of XOD in liver tissue homogenates in each group. EEGS-L: the low dose of EEGS group; EEGS-H: the high dose of EEGS group; CA: chlorogenic acid group; t-*p-*CA: trans-*p*-coumaric acid group; CA + t-*p*-CA: chlorogenic acid + trans-*p*-coumaric acid group. Data are expressed as mean ± SEM (n = 5). ***p < 0.001 versus control group; ^#^ *p* < 0.05, ^##^ *p* < 0.01 versus adenine group.

We evaluated the general appearance and histopathological changes in the livers of the rats to investigate the effects of EEGS and selected metabolites (Figures 2A–D). The liver tissues of the control group had a dark red, soft, and smooth appearance. In contrast, the liver tissues of the adenine group were bright red with a rough surface and an increased liver index (5.31% ± 0.23%). The livers treated with EEGS-H and selected metabolites (CA, t-*p*-CA, CA + t-*p*-CA) presented a soft texture, smooth surface, and a relatively dark red color, and showed a decrease in the liver index (EEGS-H: 4.24% ± 0.16%, CA: 4.27% ± 0.20%, t-*p*-CA: 4.40% ± 0.13%, CA + t-*p*-CA: 4.31% ± 0.16%; *p* < 0.05) compared with those in the adenine group. The liver tissues in the EEGS-L and allopurinol groups were slightly red. In addition, using H&E staining and inflammation scores revealed that the hepatic lobule structure disappeared, and inflammatory cell infiltration was greater in the adenine group was high (2.8 ± 0.2) than in the control group (0.4 ± 0.2). After treatment with EEGS-H and selected metabolites, the liver tissue injury was significantly repaired, with well-observed intact liver tissue structure, slight degeneration, and inflammatory infiltration (EEGS-H: 1.2 ± 0.2, CA: 1.6 ± 0.2, t-*p*-CA: 1.6 ± 0.4, CA + t-*p*-CA: 1.4 ± 0.4; *p* < 0.05).

To confirm the inhibitory effects of EEGS and selected metabolites on urate production *in vivo*, we measured liver XOD activity. As shown in Figure 2E, liver XOD activity in the adenine group (16.1 ± 0.4 U/gprot; *p* < 0.001) was significantly greater than that in the control group (8.3 ± 0.3 U/gprot). Compared with those in the adenine group, the liver XOD activities in the EEGS-H and CA + t-*p*-CA treatment groups (13.6 ± 0.7 U/gprot, 13.8 ± 0.6 U/gprot, respectively; *p* < 0.05) were significantly lower.

### 3.4 EEGS and selected metabolites alleviated adenine-induced renal injury in rats

**FIGURE 3:**
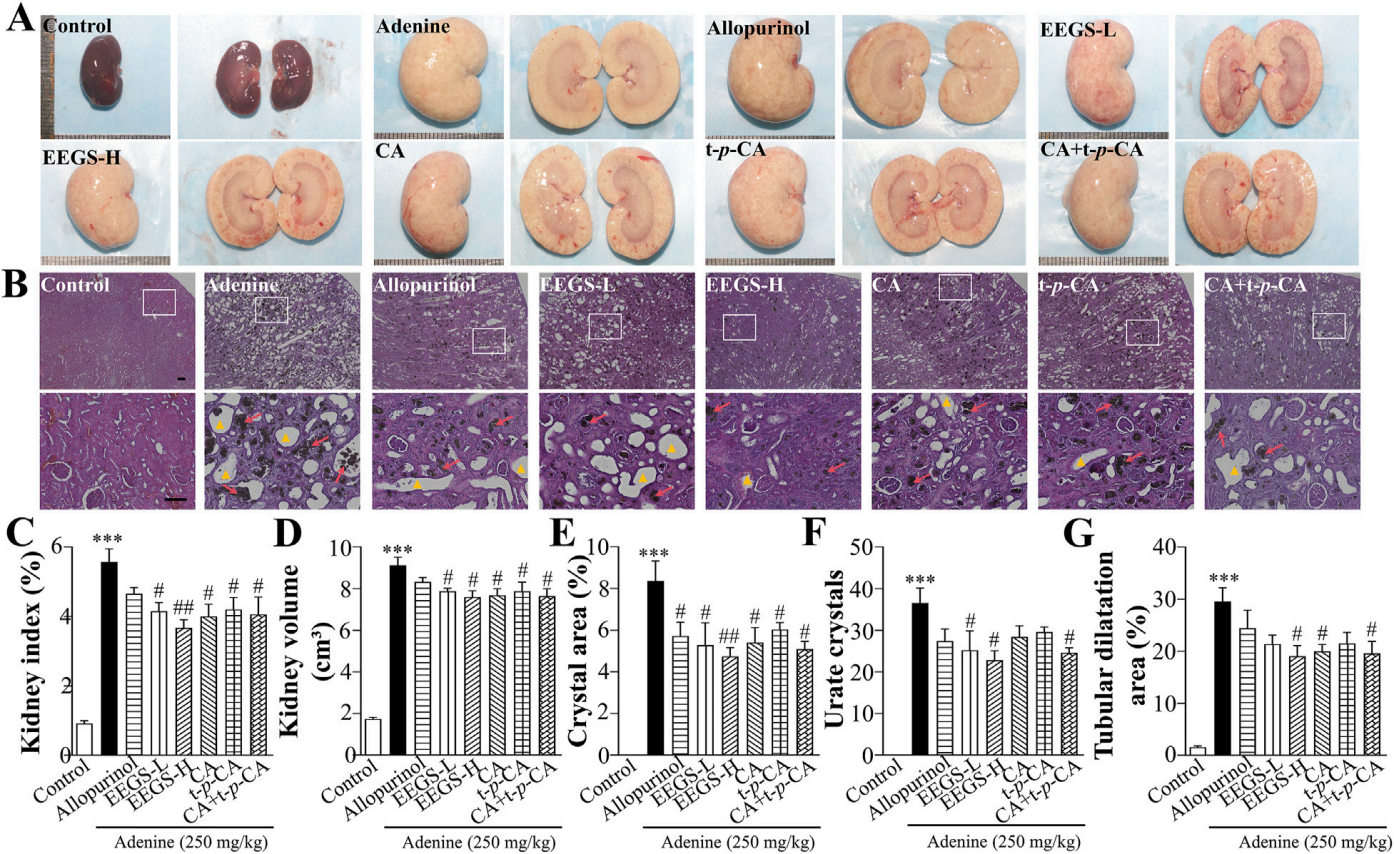
Effects of EEGS and selected metabolites intervention on adenine-induced kidney tissue in rats. **(A)** General view and cross-section of the kidneys of each group; **(B)** H&E staining images of the kidneys of each group (×4 and ×20 objective lens). The red arrows indicate urate crystals, and the triangles indicate dilatation of the tubular lumen of the kidney. The second row (×20 objective lens) is a partially enlarged view of the first-row picture (×4 objective lens). The scale bar is 100 μm for all images; **(C)** Kidney index of each group of rats; **(D)** Kidney volume of each group of rats; **(E)** Percentage area of urate crystals of kidney samples in H&E stained images; **(F)** Number of urate crystals of kidney samples in H&E stained images; **(G)** Percentage area of the tubular lumen of kidney samples in H&E stained images. EEGS-L: the low dose of EEGS group; EEGS-H: the high dose of EEGS group; CA: chlorogenic acid group; t-*p*-CA: trans-*p*-coumaric acid group; CA + t-*p*-CA: chlorogenic acid + trans-*p*-coumaric acid group. Data are expressed as mean ± SEM (n = 5). ***p < 0.001 versus control group; ^#^ *p* < 0.05, ^##^ *p* < 0.01 versus adenine group.

The control group exhibited a standard kidney shape, appearing reddish-brown with a glossy surface (Figure 3A). In contrast, the kidneys treated with adenine were significantly enlarged, pale or yellowish, with an uneven surface, bulges visible on the cut surfaces, a widened cortex, a clear boundary between the turbid cortex and medulla, and lamellar congestion and edema in the renal papillae. After treatment with EEGS-H and selected metabolites, the kidney volume decreased with a white but slightly red appearance, punctate hemorrhage, and edema. EEGS-H treatment was the most effective, and it significantly decreased the kidney index (3.7% ± 0.2%, *p* < 0.01) and volume (7.6 ± 0.3 cm^3^, *p* < 0.05; Figures 3C, D) compared with those of the adenine group (5.6% ± 0.3% for kidney index, 9.1 ± 0.4 cm^3^ for kidney volume).

The glomeruli and tubules in the control group were structurally intact, with a regular tubular lumen, no dilatation or necrosis, neatly arranged epithelial cells, an entire brush border, and no brown urate crystals (Figure 3B). In contrast, adenine treatment resulted in glomerular capsule shrinkage, compensatory expansion of the tubules, and many brown urate crystals. The infiltration of inflammatory cells into the renal interstitium was observed. The renal histopathology in the treatment group revealed fewer crystals and smaller crystalline and tubular dilatation areas than those in the adenine group (8.4% ± 0.9% for the crystal area, 36.6 ± 3.3 for urate crystals, and 29.6% ± 3.0% for the tubular dilatation area). As shown in Figures 3E–G, the EEGS-H treatment group presented significant improvements in these parameters (crystal area: 4.7% ± 0.5%, urate crystals: 22.8 ± 0.7, tubular dilatation area: 19.0% ± 2.4%; *p* < 0.05).

### 3.5 EEGS and selected metabolites reduced inflammation by improving renal interstitial fibrosis and reducing apoptosis

**FIGURE 4:**
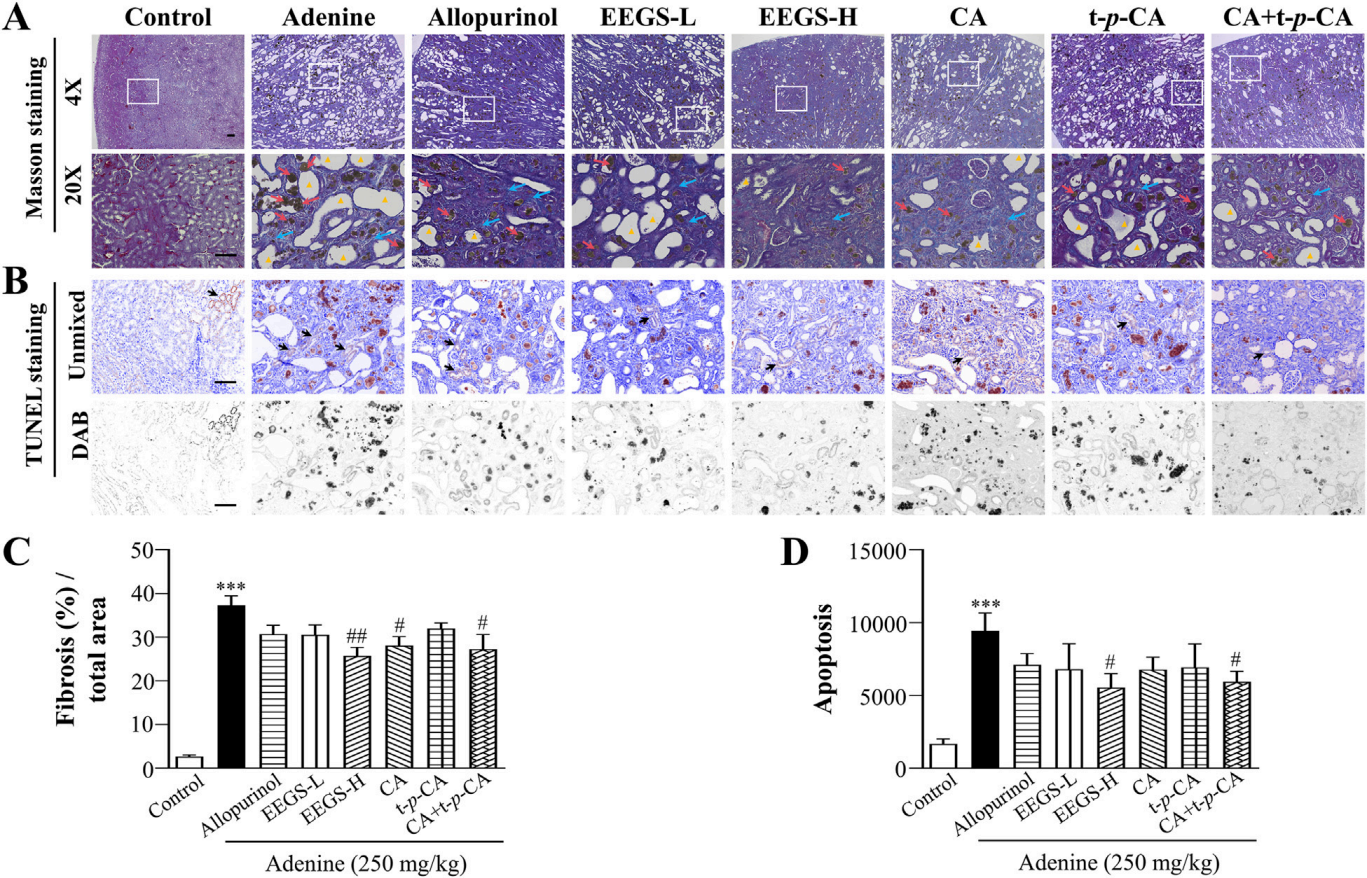
EEGS and selected metabolites attenuated renal injury by improving renal interstitial fibrosis and reducing apoptosis. **(A)** Masson staining images of rat kidneys from each group (×4 and ×20 objective lens). The blue arrows indicate renal interstitial fibers, the red arrows indicate urate crystals and the triangles indicate dilatation of the tubular lumen of the kidney. The second row (×20 objective lens) is a partially enlarged view of the first-row picture (×4 objective lens). The scale bar is 100 μm for all images; **(B)** TUNEL kit to detect apoptosis. The black arrows indicate positive expression; **(C)** Quantitative results of Masson staining of collagen fibers; **(D)** Quantitative expression of apoptosis. Data are expressed as mean ± SEM (n = 5). ***p < 0.001 versus control group; ^#^ *p* < 0.05, ^##^ *p* < 0.01 versus adenine group.

Renal anemia is inseparably linked to irreversible renal injury. Thus, we further evaluated renal injury using Masson and TUNEL staining. Adenine treatment caused numerous collagen fibers that proliferated in the renal tubular interstitium, which are marked in dark blue (Figure 4A). The quantitative results are shown in Figure 4C. The adenine group had a significantly larger area of collagen fibers area (37.3% ± 2.1%; *p* < 0.001) than did the control group (2.6% ± 0.4%). The area of collagen fibers was significantly reduced in the CA and CA + t-*p*-CA groups (28.1% ± 2.0% and 27.2% ± 3.4%, respectively; *p* < 0.05), as well as in the EEGS-H group (25.7% ± 1.9%; *p* < 0.01), and the remaining groups also showed a significant trend toward a reduced area as compared with the adenine group. In addition, as shown in Figures 4B, D, apoptosis was greatly reduced in the EEGS-H and CA + t-*p*-CA groups (5544.5 ± 949.8 and 5939.0 ± 720.8, respectively; *p* < 0.05) compared with the adenine group (9437.5 ± 1224.2).

### 3.6 EEGS and selected metabolites inhibited the expression of the inflammatory factors β-catenin and COX-2, upregulated HIF-2α expression, and promoted EPO expression

**FIGURE 5:**
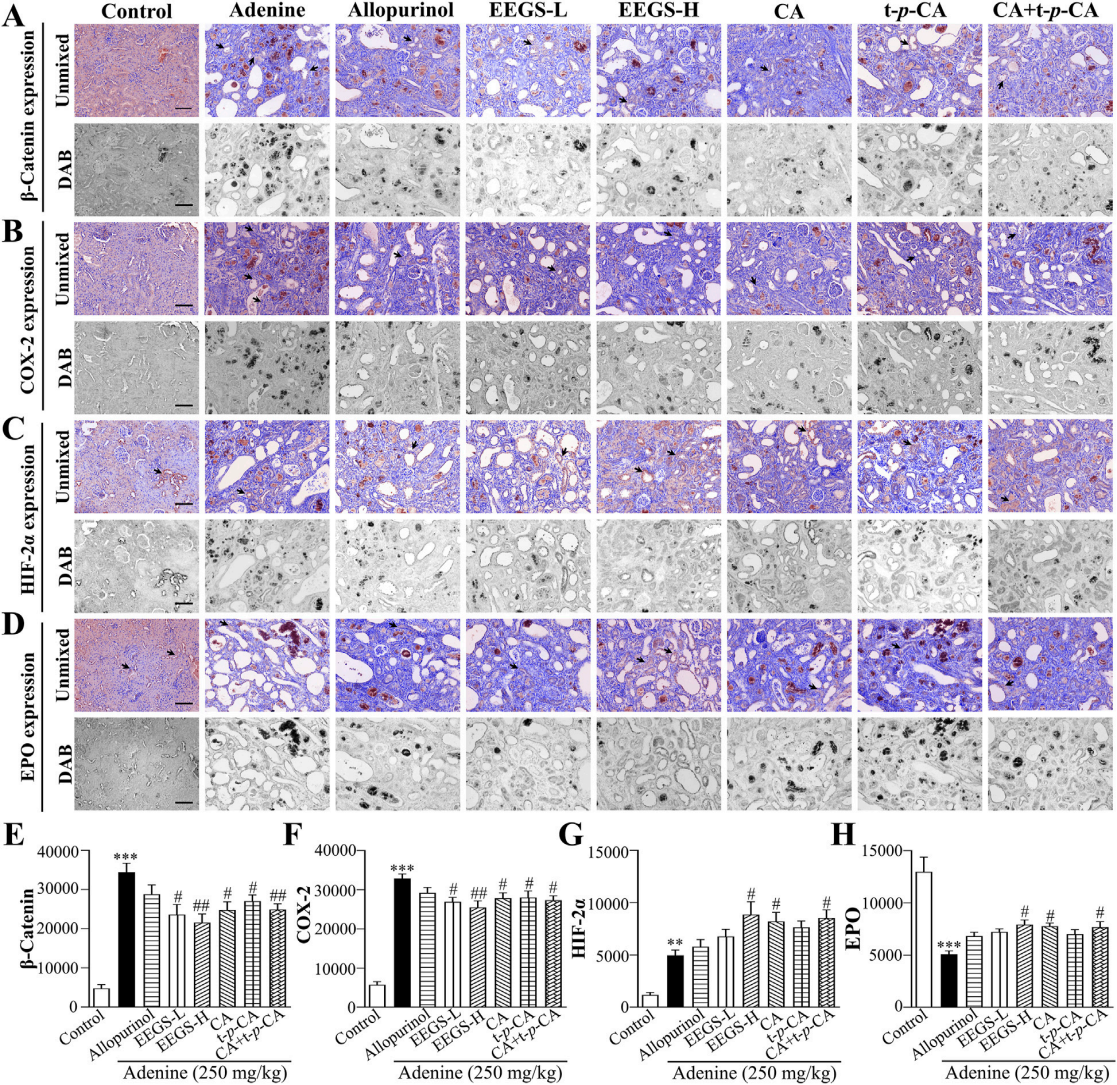
EEGS and selected metabolites inhibited the expression of inflammatory factors β-catenin and COX-2, upregulated HIF-2α expression, and promoted EPO expression **(A)** Immunohistochemical detection of β-catenin expression; **(B)** Immunohistochemical detection of COX-2 expression; **(C)** Immunohistochemical detection of HIF-2α expression; **(D)** Immunohistochemical detection of EPO expression; **(E)** Quantitative expression of β-catenin; **(F)** Quantitative expression of COX-2; **(G)** Quantitative expression of HIF-2α; **(H)** Quantitative expression of EPO. The black arrows indicate positive expression. The scale bar is 100 μm for all images (×20 objective lens). EEGS-L: the low dose of EEGS group; EEGS-H: the high dose of EEGS group; CA: chlorogenic acid group; t-*p*-CA: trans-*p*-coumaric acid group; CA + t-*p*-CA: chlorogenic acid + trans-*p*-coumaric acid group. Data are expressed as mean ± SEM (n = 5). ***p < 0.001 versus control group; ^#^ *p* < 0.05, ^##^ *p* < 0.01 versus adenine group.

We analyzed the protein expression levels of β-catenin, COX-2, HIF-2α, and EPO in the kidneys through immunohistochemistry and multispectral imaging to further investigate the mechanism of action of EEGS in correcting renal anemia (Figures 5A–H). The protein expression of EPO in the adenine group (5081.0 ± 331.5, *p* < 0.001) was significantly lower than that in the control group (12,980.8 ± 1411.4), which was consistent with the trend in the serum EPO levels. However, the EPO protein levels in the EEGS-H treatment group (7926.4 ± 436.6, *p* < 0.05) were significantly greater than those in the adenine group. Since HIF-2α is crucial for regulating EPO levels in the kidneys, we further analyzed the effects of EEGS and selected metabolites on HIF-2α. Compared with that in the control (1172.2 ± 234.9), there was a significant increase in HIF-2α expression in the adenine (4957.0 ± 511.5, *p* < 0.01). After administration of EEGS-H, CA, or CA + t-*p*-CA, HIF-2α expression increased (8851.1 ± 1234.3, 8211.7 ± 859.8, and 8527.4 ± 783.4, respectively, *p* < 0.05). We subsequently analyzed the expression of the inflammatory factors β-catenin and COX-2 in the kidneys. Compared with the adenine treatment (34,412.9 ± 2286.5 and 32,895.7 ± 1146.5), EEGS-H treatment decreased the expression of β-catenin and COX-2 (21,557.0 ± 2198.2 and 25,480.1 ± 1691.8, respectively, *p* < 0.01), increasing the expression of EPO; in contrast, allopurinol treatment did not significantly affect these parameters.

## 4 Discussion

In this study, we investigated the effects of EEGS and selected metabolites on renal anemia caused by renal injury and examined the underlying mechanisms. Our results confirmed that EEGS and selected metabolites (CA, and t-*p*-CA) could correct renal anemia by inhibiting XOD activity, reducing inflammation, and alleviating renal injury.Renal injury results in a decrease in the production of EPO, which directly results in reduced erythropoiesis, leading to anemia. This type of anemia, and is a common complication in patients with chronic kidney disease. Promoting the release of EPO or alternative therapy to return to normal erythropoiesis is an important method for correcting hematopoietic dysfunction caused by kidney injury and is also the key to treating renal anemia. Renal anemia is a specific form of chronic kidney damage characterized by impaired EPO production and decreased erythropoiesis (Shahab and Saifullah Khan, 2020). EPO is a hematopoietic growth factor that stimulates the proliferation, survival, and differentiation of erythroid progenitor cells, thereby promoting erythropoiesis (Tsiftsoglou, 2021). Although the use of erythropoietin-stimulating agents is crucial for resolving renal anemia, this therapy is not indicated in patients who exhibit poor responses to EPO or develop antibodies against it (Benjamin et al., 2020). Therefore, it is necessary to develop new and effective drugs for treating renal anemia that address the limitations of the current therapies.

The development of anemia is closely related to oxygen transport and erythropoiesis. HGB and RBCs are essential indicators of oxygen transport in the blood, and when a decrease in HGB or RBC levels to below the normal range results in anemia. We observed that weight loss and anemia occurred in adenine-induced CKD rats, and that the administration of EEGS-H improved these outcome. Among the EEGS selected metabolites, the combined effect of CA and t-*p*-CA improved anemia more effectively than either compound alone. In addition, the SCr level can accurately reflect glomerular filtration function, which is crucial for diagnosing and treating CKD (Nah et al., 2016). Additionally, it serves as a clinically useful indicator of renal function, and elevated SCr levels in adenine-treated rats indicate decreased kidney function (Gorelik et al., 2022). However, EEGS and selected metabolites reversed these abnormal changes in SCr and improved the kidney function.

Pathological examinations revealed that many needle-like crystals and tubular lumen dilatations were present in the adenine-treated renal tissues of the rats, which exacerbated the progression of renal injury and renal anemia. The crystals appeared not only in the renal tubules but also in the renal interstitium and glomeruli. Smaller crystals are degraded by renal tubular epithelial cells through endocytosis; in contrast, larger crystals clog the entire renal tubule and overgrow in the presence of macrophages, which transfer the crystals to other parts of the tissue (Klinkhammer et al., 2020). These crystals cause a localized chronic inflammatory response and oxidative stress. Therefore, we chose allopurinol, a clinically used drug to inhibit XOD activity, as the tool drug for this study (Bove et al., 2017). Although allopurinol inhibited XOD activity by 93.56% *in vitro*, no reduction in inflammation or liver/renal injury was observed *in vivo*, nor was the alleviation of anemia observed, which is consistent with its inability to prevent the development of CKD (Badve et al., 2020). Notably, EEGS inhibited XOD activity *in vitro* by 88.53%, which was lower than that of allopurinol. However, EEGS significantly inhibited liver XOD activity, reduced the number of crystals in the kidney, and reversed liver and renal injury, thereby ameliorating anemia. This finding is consistent with the literature which reports that EEGS has a high antioxidant activity (Tan et al., 2016).

As one of the crucial active constituents of EEGS, CA has good therapeutic effects, and its combination with t-*p*-CA is even more effective. CA is a polyphenolic compound that inhibits XOD activity and is used to treat kidney diseases associated with oxidative stress and inflammatory processes (Ferraz-Filha et al., 2017). T-*p*-CA is another important bioactive substance in EEGS that has anti-inflammatory and antioxidant properties (Venkatesan et al., 2023). When free adenine cannot be broken down in the liver, hepatic XOD activity increases, which subsequently leads to liver and renal damage (Pritsos, 2000). Our results suggest that the treatment with a combination of CA and t-*p*-CA could attenuate oxidative stress damage caused by purines, significantly inhibit XOD activity, ameliorate liver and kidney injury, and effectively correct anemia.

When the kidneys are subjected to sustained injury, renal interstitial cells inevitably move toward interstitial fibrosis, and the production of EPO decreases (Zhu et al., 2012). Many EPO-producing cells transform into myofibroblasts, resulting in inadequate the EPO production and exacerbation of anemia (Kobayashi et al., 2022). In chronic diseases, kidney enlargement is thought to result from a fibrotic response (Luo et al., 2021). We found that by attenuating collagen fibrosis and inhibiting kidney enlargement, EEGS and selected metabolites increased EPO synthesis, thereby ameliorating anemia. In recent years, researchers have often combined other methods to verify the presence of fibrosis to increase the credibility and comprehensiveness of their studies. Some researchers have observed renal fibrosis by Sirius Red staining for collagen deposition and immunohistochemical staining to detect molecular markers such as TGF-β1 and α-SMA, which is consistent with the collagen deposition observed by Masson staining in this study (Jiao et al., 2021a).

The regulation of renal tubular apoptosis is essential for maintaining normal renal function and erythropoiesis. Apoptosis of a large number of abnormal cells leads to impaired renal tubular function, induces inflammation and tissue damage, restricts primitive erythrocyte differentiation and proliferation, and shortens the lifespan of erythrocytes (Tu et al., 2014). EEGS and selected metabolites can prolong the lifespan of erythrocytes by reducing the expression degree of apoptosis.

Recent studies have shown that the initiation and development of an inflammatory response usually causes the polarisation of macrophages, which releases inflammatory mediators. In a prolonged inflammatory state, they cause oxidative stress damage. Oxidative stress triggers anemia by producing reactive oxygen species (ROS) and reactive nitrogen species (RNS), which directly damage erythrocytes and decrease erythrocyte numbers, affecting hemoglobin production (An et al., 2023; Jiao et al., 2021b).

Inhibiting inflammation and stabilizing hypoxic signaling are also critical factors for improving renal injury. Inflammatory cytokines can increase phagocytosis by macrophages and increase erythropoietin resistance to inhibit erythropoiesis (Ganz, 2018). Under pathological hypoxic conditions, activated inflammatory signaling degrades HIF and impairs EPO production (Souma et al., 2016). β-Catenin and COX-2 mainly regulate inflammatory responses; they are expressed at low levels in normal adult kidneys but are highly expressed in inflammatory diseases, damaging the kidneys and exacerbating anemia (Zhao et al., 2019). HIF-2α is the primary regulator of EPO and is responsible for managing renal hypoxia and promoting EPO production (Haase, 2013). Our results revealed that the expression of HIF-2α and EPO was upregulated and that the high expression of inflammatory factors, including the β-catenin and COX-2 proteins, was downregulated in the kidneys after EEGS and selected metabolite treatment. Therefore, we speculated that the potential function of EEGS in correcting renal injury and anemia may be due to the increase in the HIF-2α protein level and the inhibition of inflammatory factors. This result is consistent with the finding that the expression of proinflammatory cytokines is reduced by increasing the accumulation of the HIF-2α protein (Wang et al., 2018). Thus, EEGS and selected metabolites can ameliorate renal anemia by inhibiting the overexpression of β-catenin and COX-2 and enhancing the expression of HIF-2α.

Our study revealed that EEGS has unique advantages in the treatment of renal anemia, especially in inhibiting the activity of XOD and reducing the production of crystals in the kidneys. Currently, there are limitations to the commonly used clinical drugs for the treatment of renal anemia, such as erythropoietin (EPO) and iron. For example, erythropoietin may cause adverse effects such as hypertension and thrombosis, whereas long-term use of iron may lead to gastrointestinal discomfort or excessive accumulation (Stevens et al., 2024). Compared with these drugs, EEGS has a more comprehensive protective effect on the liver and kidneys and can potentially be an alternative drug for treating renal anemia. EEGS, because of its multiple biological effects such as anti-inflammatory and antioxidant effects, may be useful in improving the symptoms of diseases, such as liver injury and blood dysfunction.

Our preliminary study revealed that each component alone was less effective for treating renal anemia than their combination or EEGS, suggesting a limitation. Therefore, we believe that one or more of the components in EEGS, other than CA and t-*p*-CA, may ameliorate renal injury and correct anemia or augment the activity of these two constituents. Moreover, multiple pathways, including those regulating iron metabolism, are also involved in improving renal anemia. Therefore, further studies are needed to better understand these mechanisms.

## Data Availability

The original contributions presented in the study are included in the article/supplementary material, further inquiries can be directed to the corresponding authors.
